# Concomitant attenuation of HMG-CoA reductase expression potentiates the cancer cell growth-inhibitory effect of statins and expands their efficacy in tumor cells with epithelial characteristics

**DOI:** 10.18632/oncotarget.25448

**Published:** 2018-06-29

**Authors:** Takuro Ishikawa, Yoshinao Z. Hosaka, Colin Beckwitt, Alan Wells, Zoltán N. Oltvai, Katsuhiko Warita

**Affiliations:** ^1^ Laboratory of Basic Veterinary Science, United Graduate School of Veterinary Science, Yamaguchi University, Yamaguchi 753-8515, Japan; ^2^ Department of Veterinary Anatomy, School of Veterinary Medicine, Tottori University, Tottori 680-8553, Japan; ^3^ Department of Pathology, University of Pittsburgh, School of Medicine, Pittsburgh, PA 15213, USA; ^4^ Department of Computational & Systems Biology, University of Pittsburgh, School of Medicine, Pittsburgh, PA 15260, USA

**Keywords:** cell metabolism, statin, metastasis, combination therapy, cancer therapy

## Abstract

HMG-CoA reductase (HMGCR) inhibitors, statins, are potent cholesterol reducing drugs that exhibit anti-tumor effects *in vitro* and in animal models, including attenuation of metastasis formation, and their use correlates with reduced cancer-specific mortality in retrospective human cohort studies. However, E-cadherin expressing epithelial- and mixed epithelial-mesenchymal cancer cell lines (reflective of primary and outgrowing metastatic tumor cells, respectively) require higher statin concentrations than mesenchymal-like tumor cells (reflective of in-circulation metastatic tumor cells) to achieve the same degree of growth inhibition. Here, we show that attenuation of HMGCR expression in the presence of atorvastatin leads to stronger growth inhibition than dual target blockade of the mevalonate pathway in relatively statin resistant cell lines, mainly through inhibition of protein prenylation pathways. Thus, combined inhibition of the mevalonate pathway's rate-limiting enzyme, HMGCR, can improve atorvastatin's growth inhibitory effect on epithelial- and mixed mesenchymal-epithelial cancer cells, a finding that may have implications for the design of future anti-metastatic cancer therapies.

## INTRODUCTION

Metastases are the cause of death in most cancer patients. Often, a tumor metastasizes even before the discovery of histologically encapsulated primary tumors [1, 2], meaning that the primary tumor is often found too late to prevent escape and dissemination. For example, in the 80-90% of breast cancer cases in which tumors are found and physically removed as seemingly singular nodules, 10-30% of patients suffer a relapse with metastases found at remote sites [1]. Half of those recurrences emerge more than five years after the apparent “cure” of the disease [3].

Conceptually, the formation of cancer metastases can be divided into distinct phases [4]. During the first phase, a fraction of epithelial, E-cadherin (E-cad) expressing primary tumor cells undergo partial or complete epithelial-to-mesenchymal transition (EMT) including downregulation of E-cad expression [5]. This allows them to detach from the primary tumor (as single cells or cell clusters) [6], migrate through a barrier matrix, and intravasate into the circulation. Some of these circulating cells survive the transit and successfully colonize distant organs by partially reverting to an epithelial phenotype through mesenchymal-to-epithelial reverting transition (MErT) [7]. These cells often enter a phase of quiescent dormancy [8, 9], during which they are in intimate communication with the non-transformed microenvironment [7]. This phase is of variable length and is followed by an outgrowth phase, in which the initially dormant micrometastases expand in size, which requires at least a partial EMT [4].

Unfortunately, chemotherapy is far more effective against primary tumors than against clinically evident metastases or micrometastases. Therefore, therapies that target micrometastases by either attenuating their formation, cytotoxic killing, or maintaining cells in a dormant state are highly desired to prolong patient survival. However, traditional cancer therapies target rapidly dividing cells. As these micrometastases are either non-proliferative or slowly growing, effective new compounds are needed that function through a different mechanism of action.

**Figure 1:**
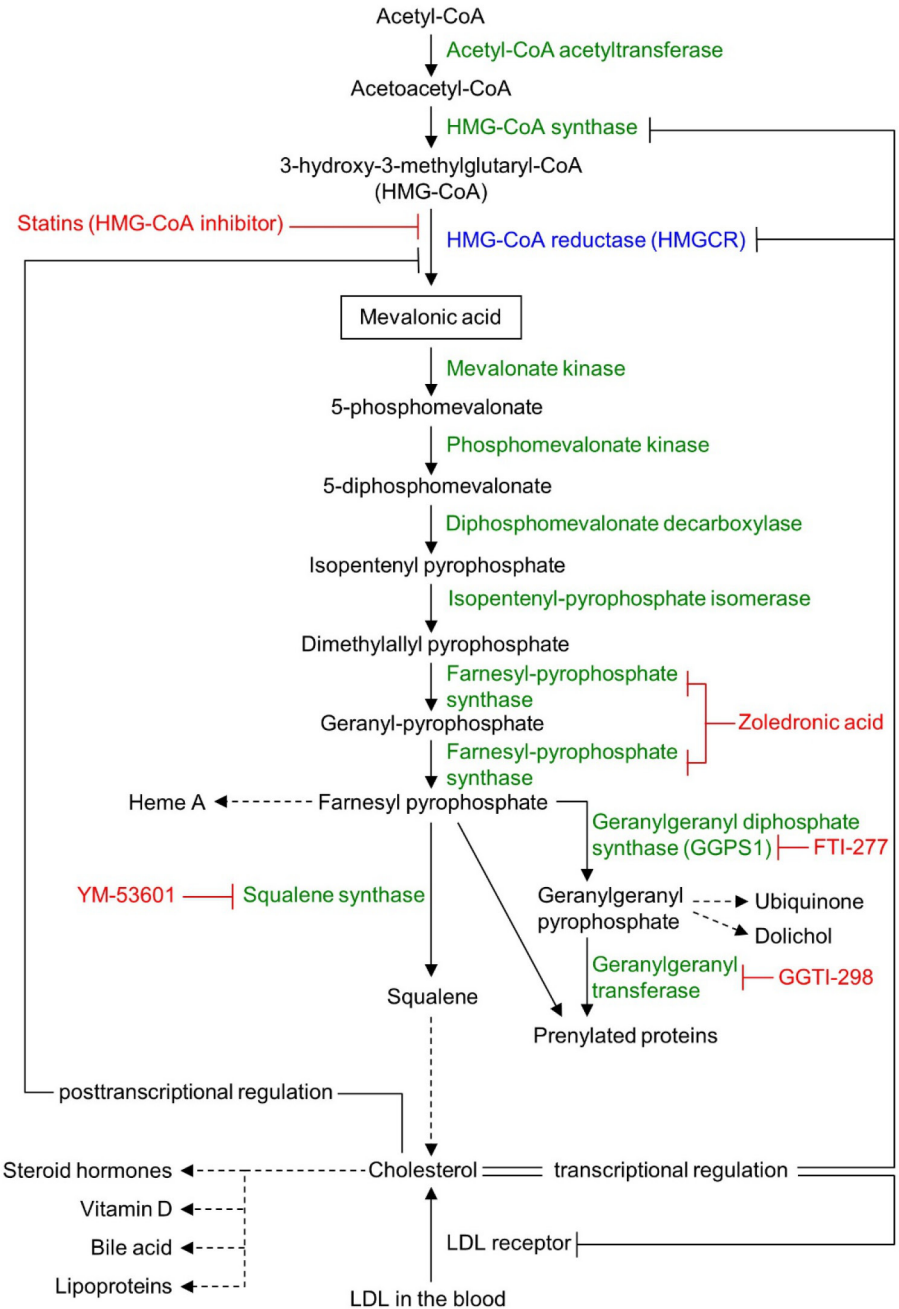
Schematic view of the mevalonate pathway Mevalonate pathway enzymes are shown in green (HMG-CoA reductase [HMGCR] in blue) and their chemical inhibitors are depicted in red. Dashed arrows represent multi-enzyme processes.

Statins specifically inhibit the activity of the rate-limiting enzyme, HMGCR, of the mevalonate pathway (Figure 1) and are used for the treatment of hypercholesterolemia by millions of people worldwide. Statins can also suppress cancer cell proliferation [10–12], cancer stem cells [13, 14], migration and invasiveness [15] and metastases formation in murine tumor models [16, 17]. Statin induced inhibition of HMGCR also decreases the levels of mevalonate and its downstream products, including cholesterol and the isoprenoid intermediates farnesyl (FPP)- and geranylgeranyl-pyrophosphate (GGPP) (Figure 1). Geranylgeranyl transferase inhibitors mimic the effect of statins on some tumor cell lines, while farnesyl transferase inhibitors are less effective [10].

In statin-sensitive cell lines, the inhibition of HMGCR reduces cell growth by different mechanisms. Parenthetically, it must be noted that ‘sensitive’ and ‘resistant’ are relative terms implying left- or right-shifted dose response curves, respectively. First, decreasing the activity of Rho GTPase (itself dependent on the mevalonate pathway-produced metabolite, GGPP) [15, 18] inactivates the transcription factors (TFs) YAP and TAZ of the Hippo pathway [19]. These TFs are crucial for normal organ size control and stem cell renewal, but also play an important role in tumorigenesis and metastasis formation in their deregulated state [19] by inducing mesenchymal differentiation [20], cancer stem cell traits [21], and cancer cell motility [15]. Second, the activity of canonical MEK/Erk and PI3-kinase/Akt/mTor signaling pathways are dependent on membrane-anchored prenylated Ras activity that is inhibited by statins through GGPP depletion [22]. Finally, statins may also impair the glucose uptake of tumor cells [23]. This effect may relate to the lower concentration of cholesterol in the cell membrane, which impairs membrane lipid raft functions, and the subcellular localization and function of glucose transporters [23], and other receptor complexes [24]. Accumulation of metabolic precursors such as acetyl CoA could also block glucose uptake through feedback inhibition of glycolysis [25]. The latter two mechanisms target the glycolytic nature of tumor cells, thereby also providing for a therapeutic index to minimize toxicities. These findings imply that inhibition of other pathways may potentiate statin effects.

We and others have demonstrated earlier that statins preferentially attenuate the proliferation of slow growing, mesenchymal-like cancer cell lines *in-vitro* [26–28]. Importantly, cells that leave the primary tumor, and colonize other organs display exactly this phenotype. In turn, after a period of dormancy, growth-reactivated micrometastases display mixed epithelial-mesenchymal phenotypes [4, 7]. Previous studies have shown that mixed epithelial-mesenchymal and purely epithelial cells are relatively more resistant to statin-mediated growth suppression than mesenchymal-like tumor cells [26–28]. Moreover, even statin-sensitive cell lines require statins at a concentration that is an order of magnitude higher than observed in human plasma during standard hypercholesterolemia therapy [27, 29]. Thus, there is a significant clinical need to identify existing drugs or novel compounds that could enhance the effect of statins on cancer cells. Such compounds may also provide a mechanistic rationale for using statin combination therapies as an adjuvant cancer treatment or for delaying metastasis development.

Here, we examine the role of mevalonate pathway reactions downstream from mevalonic acid production and the effect of different type of combination therapies on potentiating atorvastatin's growth inhibitory effect in statin-resistant cells lines. We show that statins inhibit the growth of cancer cell lines mainly through inhibition of protein prenylation pathways and that attenuation of HMGCR mRNA and protein expression in the presence of atorvastatin provides much stronger growth inhibitory effect on relatively statin resistant cell lines than inhibiting two enzymes of the mevalonate pathway. Thus, combined inhibition of HMGCR can improve statin sensitivity of epithelial and mixed mesenchymal-epithelial cancer cells.

## RESULTS

### Statins exerts their growth inhibitory effects through blocking HMG-CoA reductase

We have shown previously that the sensitivity of cancer cell lines to statins’ growth inhibitory effect varies significantly, ranging from highly statin sensitive mesenchymal- to less statin sensitive epithelial and mixed epithelial-mesenchymal cells [27, 30]. The differential effect of statins on cancer cells may be due to different effects on the expression or subcellular distribution of their target enzyme, HMGCR (Figure 1), or due to additional off-target effects of statins. Indeed, higher HMGCR levels are associated with atorvastatin resistance in breast cancer [31]. However, our previous study revealed that the fourteen cancer cell lines we have studied, including the epithelial NCI-H332M, mixed mesenchymal-epithelial DU-145, and mesenchymal PC-3 and HOP-92 cell lines (Supplementary Figure 1A-1D) express HMGCR at comparable levels under normal growth conditions [27]. To test if HMGCR levels were affected by statin therapy, we examined its expression in one of the statin-resistant (DU-145) cancer cells at atorvastatin concentrations below their respective IC_50_ values. In agreement with previous results [32], we observed an upregulation of HMGCR mRNA levels in DU-145 cells that was proportional to the concentration of atorvastatin in the growth medium (Supplementary Figure 2A), yet HMGCR protein expression levels did not significantly change upon 24 hours or 48 hours of atorvastatin treatment (Supplementary Figure 2B, 2D). As reported previously [33], HMGCR levels are maintained by the feedback response that upregulates both HMGCR mRNA and low-density lipoprotein (LDL)-receptors (LDLR) that enables cholesterol uptake from the serum-containing media; thus alteration in HMGCR protein is not evident as cholesterol homeostasis has been achieved, even in response to statins that do trigger an anti-proliferative response. Treatment with another statin, rosuvastatin, which does not inhibit the growth of DU-145 cells [30], yielded the same result (Supplementary Figure 2C, 2E).

Altered HMGCR subcellular localization may also contribute to statin resistance. To test this hypothesis, we next examined the HMGCR expression patterns in PC-3, DU-145, HOP-92 and NCI-H322M cells before and after atorvastatin therapy. Immunostaining for HMGCR, an integral ER membrane protein [34], revealed that the enzyme displays a largely perinuclear cytoplasmic distribution in all four cell lines (Supplementary Figure 3A). This distribution does not change after 12-36 hours of atorvastatin treatment either in statin-sensitive (Supplementary Figure 3B) or resistant cell lines (Supplementary Figure 3C). We also compared HMGCR's subcellular localization with that of the ER marker protein, CellLight ER-RFP, 24 hours after vehicle control or atorvastatin treatment. We find that in statin sensitive cells (HOP-92, PC-3) there is no alteration in HMGCR expression magnitude nor in the relationship to the ER signal after statin treatment (Supplementary Figure 3D).

**Figure 2:**
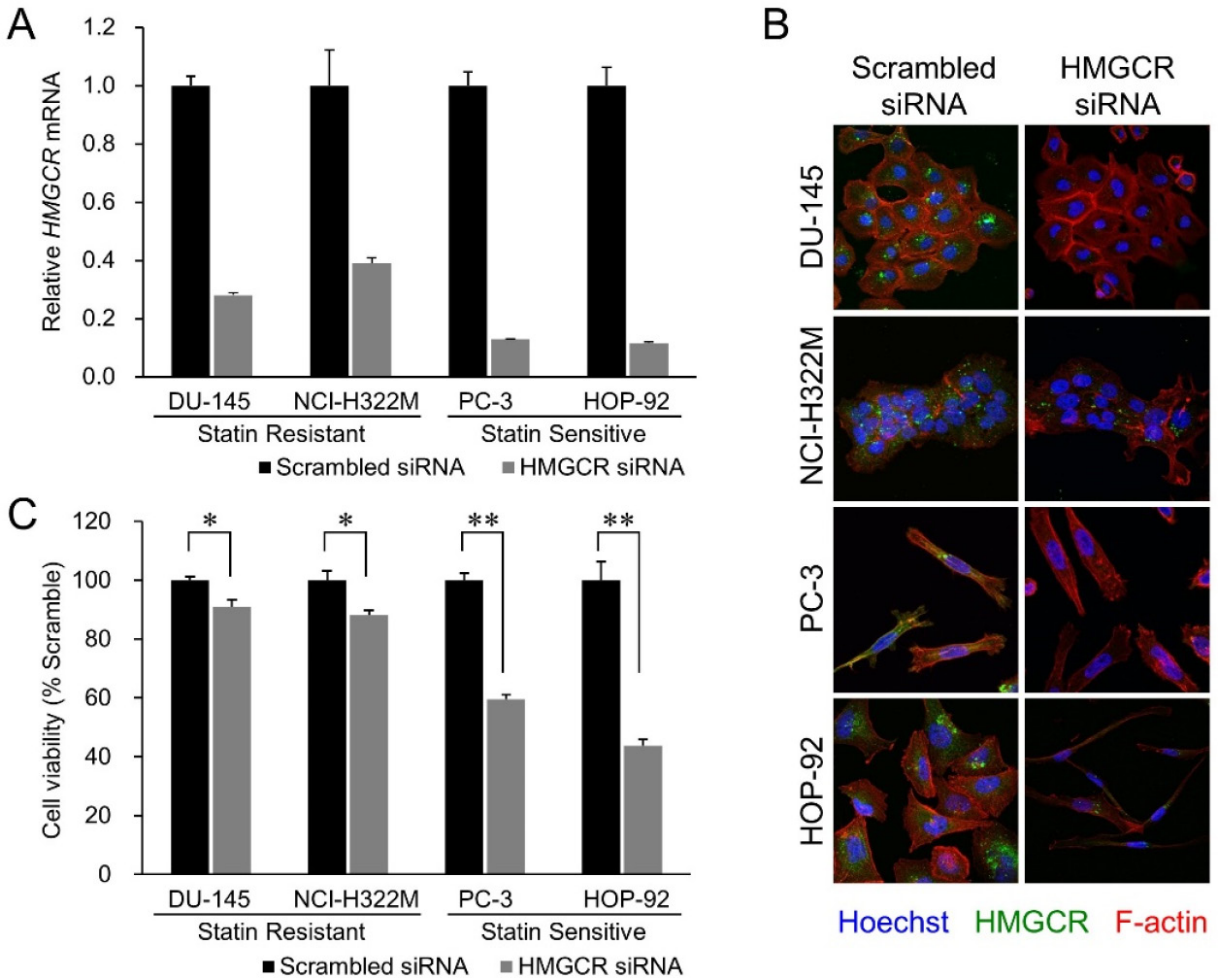
HMGCR knockdown recapitulates statin's growth inhibitory effect **(A)** Effect of siRNA transfection on endogenous *HMGCR* mRNA levels in the four cell lines. The cells were transfected with 10 nM of siRNAs targeting *HMGCR* or with their scrambled versions (control). Samples were analyzed 72 hours after the beginning of transfection. Data were normalized to the *GAPDH* mRNA levels in each sample and expressed in terms of a value relative to the control. Each column represents the mean ± SD (n = 3 for each group with triplicate determination). **(B)** Merged images of the siRNA-transfected cell lines immunostained for HMGCR (green, perinuclear), F-actin (red, cytoplasmic), and Hoechst (blue, nucleus). HMGCR immunoexpression was diminished by HMGCR siRNA treatment. In addition, remarkable cellular shrinkage was observed in the HMGCR knockdown-HOP-92 group. **(C)** Cell viability of the statin-resistant cell lines (DU-145 and NCI-H322M) and -sensitive cell lines (PC-3 and HOP-92) treated with HMGCR siRNA 72 hours after the beginning of transfection. Values in scrambled control were set to 100%. Each value represents the mean ± SD (n = 3). Data were analyzed using a student's two-tailed t-test: ^*^ p < 0.05, ^**^ p < 0.01.

We next examined if atorvastatin exerts its growth-inhibitory effect on cancer cell lines by selectively inhibiting HMGCR or also by off-target effects. Inhibition of *HMGCR* expression with *HMGCR*-specific siRNA-mediated knockdown substantially reduced both *HMGCR* mRNA (Figure 2A) and HMGCR protein expression (Figure 2B) and phenocopied atorvastatin's growth inhibitory effect in both statin resistant (DU-145, NCI-H322M) and sensitive (HOP-92, PC-3) cell lines (Figure 2C). We and others have also demonstrated previously that the addition of mevalonic acid, the metabolic substrate produced by the enzymatic activity of HMGCR (Figure 1), countered the growth inhibitory effects of atorvastatin in statin-sensitive cells [26–28]. These data thus imply that statins exert their growth inhibitory effect on cancer cell lines through their inhibition of HMGCR enzyme activity.

### Statins inhibit cancer cell proliferation through their effect on protein prenylation

**Figure 3:**
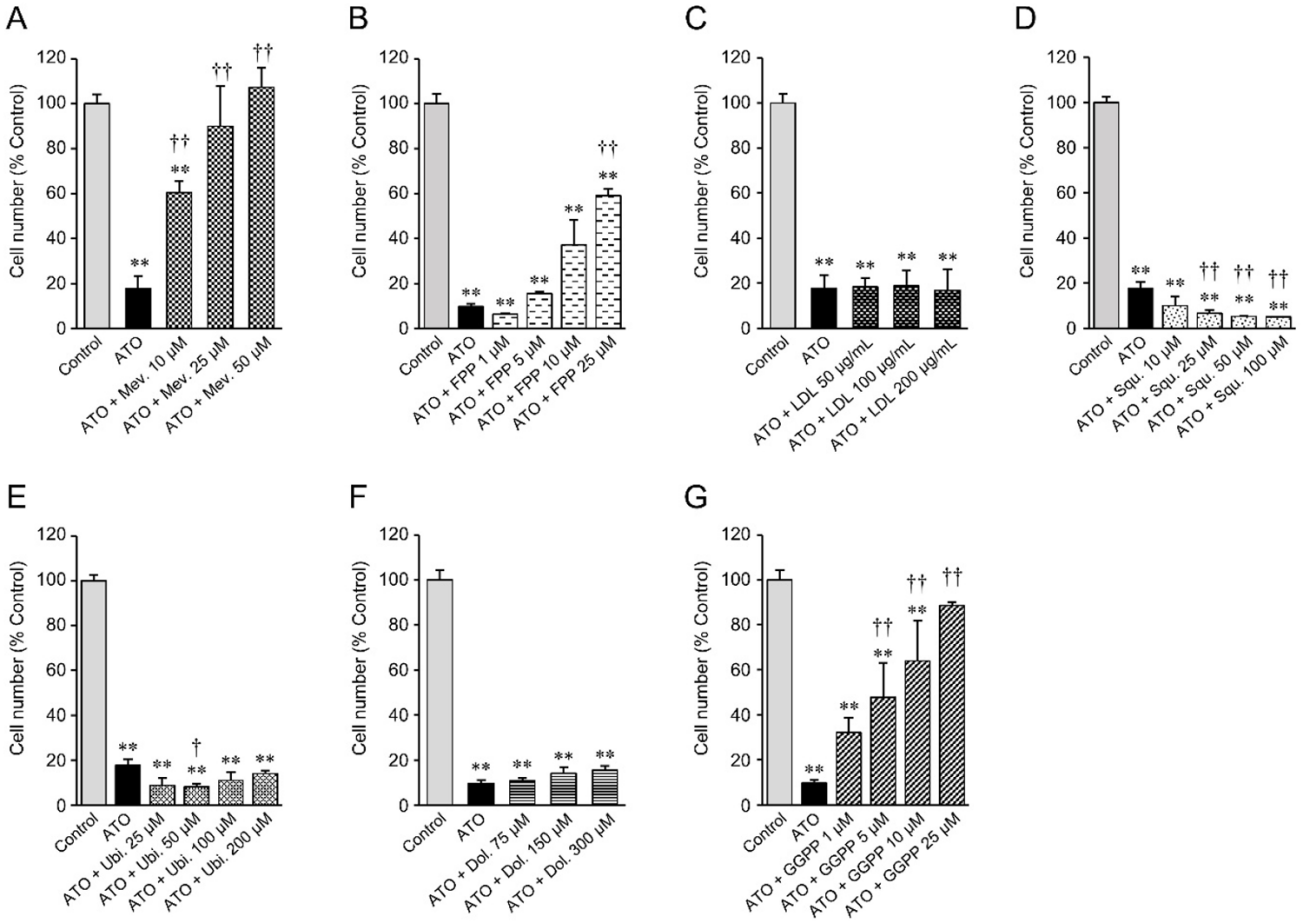
Cell number of statin-sensitive HOP-92 cells treated with atorvastatin and various intermediate metabolites of the mevalonate pathway 10 μM atorvastatin (ATO)-treated HOP-92 cells were incubated with **(A)** mevalonate, **(B)** farnesyl pyrophosphate, **(C)** LDL, **(D)** squalene, **(E)** ubiquinone, **(F)** dolichol, and **(G)** geranylgeranyl pyrophosphate at the indicated concentrations. Cell viability was measured at 48 hours after treatment. Cell viability of vehicle-treated control was regarded as 100%. Measurement values for each group were compared using the Bonferroni-Dunn *post-hoc* tests. Mean ± SD (*n* = 3) ^**^ p < 0.01 Comparison against control, ^†^ p < 0.05, ^††^ p < 0.01 Comparison against ATO-treated positive cells. Mev., mevalonate; FPP, farnesyl pyrophosphate; LDL, low-density lipoprotein; Squ., squalene; Ubi., ubiquinone; Dol., dolichol; GGPP, geranylgeranyl pyrophosphate.

The mevalonate pathway divides into several pathways after its farnesyl pyrophosphate (FPP) synthesis step (Figure 1), suggesting that one or more of the furcated pathways may be responsible for maintaining growth and proliferation in cancer cell lines. To test this hypothesis we next determined the ability of various exogenous downstream mevalonate pathway products to restore cell growth in the presence of atorvastatin, which we used at an 80% killing efficiency in order to identify those substrates that are most potent in their rescue function. As seen before, exogenous mevalonate completely rescued the growth of atorvastatin treated, statin-sensitive HOP-92 (Figure 3A, Supplementary Figures 5, 6) and PC-3 cells (Supplementary Figure 6), while the addition of FPP provided partial rescue (Figure 3B). Importantly, mevalonate or FPP supplementation does not result in enhanced cell growth (Supplementary Figure 4A, 4B).

Statins reduce blood cholesterol levels by inducing cholesterol scavenging from the circulation, in the form of lipoprotein particles, through upregulation of cell surface LDLR [35] (Figure 1). Cancer cells may similarly be able to use this mechanism to counter HMGCR inhibition. However, we found that exogenous LDL did not rescue the growth of atorvastatin-treated HOP-92 (Figure 3C, Supplementary Figures 5, 6) and PC-3 cells (Supplementary Figure 6), and the addition of squalene, an intermediate metabolite of the cholesterol synthesis pathway (Figure 1) (or added ubiquinone and dolichol), proved equally ineffective (Figure 3D-3F), while having no significant effect by themselves on cell growth (Supplementary Figure 4C-4F).

We also assessed the effect of attenuated LDLR activity on the four cell lines’ statin sensitivity by siRNA-mediated knockdown of their LDLR expression. We find that knockdown with siRNA substantially reduces *LDLR* mRNA (Supplementary Figure 7A) and LDLR protein expression (Supplementary Figure 7B) in all four cell lines without affecting their growth and proliferation (Supplementary Figure 7C). The atorvastatin sensitivity of the four cell lines were equally unaffected by reduced LDLR expression (Supplementary Figure 8). These data thus indicate that the FPP to cholesterol synthesis pathway (Figure 1) does not mediate the growth inhibitory effect of statins in these cancer cell lines.

**Figure 4:**
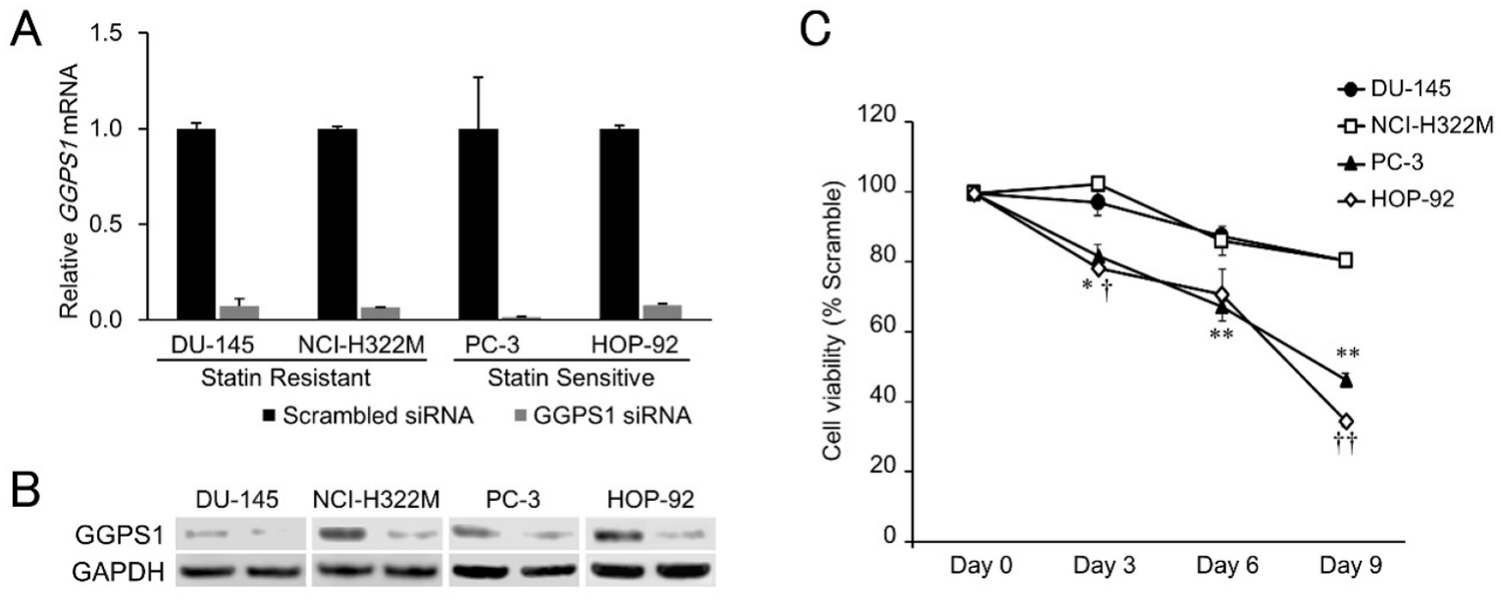
Effects of GGPS1 knockdown on statin-resistant and -sensitive cells **(A)** Expression of *GGPS1* mRNA was decreased after siRNA transfection in all four cell lines tested, as determined by RT-PCR. Data were normalized to the *GAPDH* mRNA levels in each sample and expressed in terms of a value relative to the control. Each column represents the mean ± SD (n = 3). **(B)** Protein expression of GGPS1 was decreased after siRNA transfection as determined by western blotting. Left lanes; scrambled control, Right lanes; GGPS1 siRNA-treated cells. **(C)** Cell viabilities of the statin-resistant (DU-145, NCI-H322M) and -sensitive (PC-3, HOP-92) cell lines treated with GGPS1 siRNA over a 9-day period after the beginning of transfection. Values in scrambled control were set to 100%. Each value represents the mean ± SD (n = 3). Data were analyzed using a student's two-tailed t-test: ^*^ p < 0.05, ^**^ p < 0.01 (PC-3 vs. DU-145 at the same point in time), † p < 0.05, †† p < 0.01 (HOP-92 vs. NCI-H322M at the same point in time).

Previous reports have strongly implicated the inhibited prenylation and subsequent cytoplasmic retention of small GTPase proteins Rho and Ras as the main mechanism of statins’ growth inhibition of cancer cells that is countered by exogenous geranylgeranyl pyrophosphate (GGPP) (Figure 1) [22, 36, 37]. Confirming these findings, we also found that the addition of GGPP provided near complete rescue for statin-treated HOP-92 cells (Figure 3G) while GGPP alone did not alter their overall cell number (Supplementary Figure 4G). In complementary studies, we have found that siRNA-mediated knockdown of geranylgeranyl diphosphate synthase 1 (GGPS1), the enzyme that catalyzes the synthesis of GGPP from FPP and isopentenyl diphosphate, substantially reduced *GGPS1* mRNA (Figure 4A) and protein expression (Figure 4B). As reported before [38], knockdown of GGPS1 qualitatively recapitulated the growth inhibitory effect of atorvastatin in both statin resistant and sensitive cell lines (Figure 4C).

### Inhibition of HMGCR expression potentiates atorvastatin's growth inhibitory effect

Epithelial and mixed epithelial-mesenchymal cancer cell lines are growth inhibited by atorvastatin but only at a drug concentration that is substantially higher than required for growth inhibition of mesenchymal-like tumor cells [27]. Combination therapies are beneficial because they allow similar efficacy with lower drug concentrations than used for monotherapy. As such, we wished to assess if different modes of statin combination therapies can improve the growth inhibition of the relatively more statin resistant epithelial and mixed epithelial-mesenchymal cancer cells that express E-cadherin (E-cad) on their cell membrane [27]. Indeed, interaction of mixed epithelial-mesenchymal DU-145 cells with liver cells increases their E-cad expression and resistance to cell death [39]. In turn, forced membrane expression of E-cad in the (E-cad non-expressing) mesenchymal breast cancer cell line, MDA-MB-231, significantly attenuates atorvastatin's growth inhibition [27], while inhibition of E-cad driven PI3K signaling through Akt potentiates the same cell line's statin sensitivity [37].

**Figure 5:**
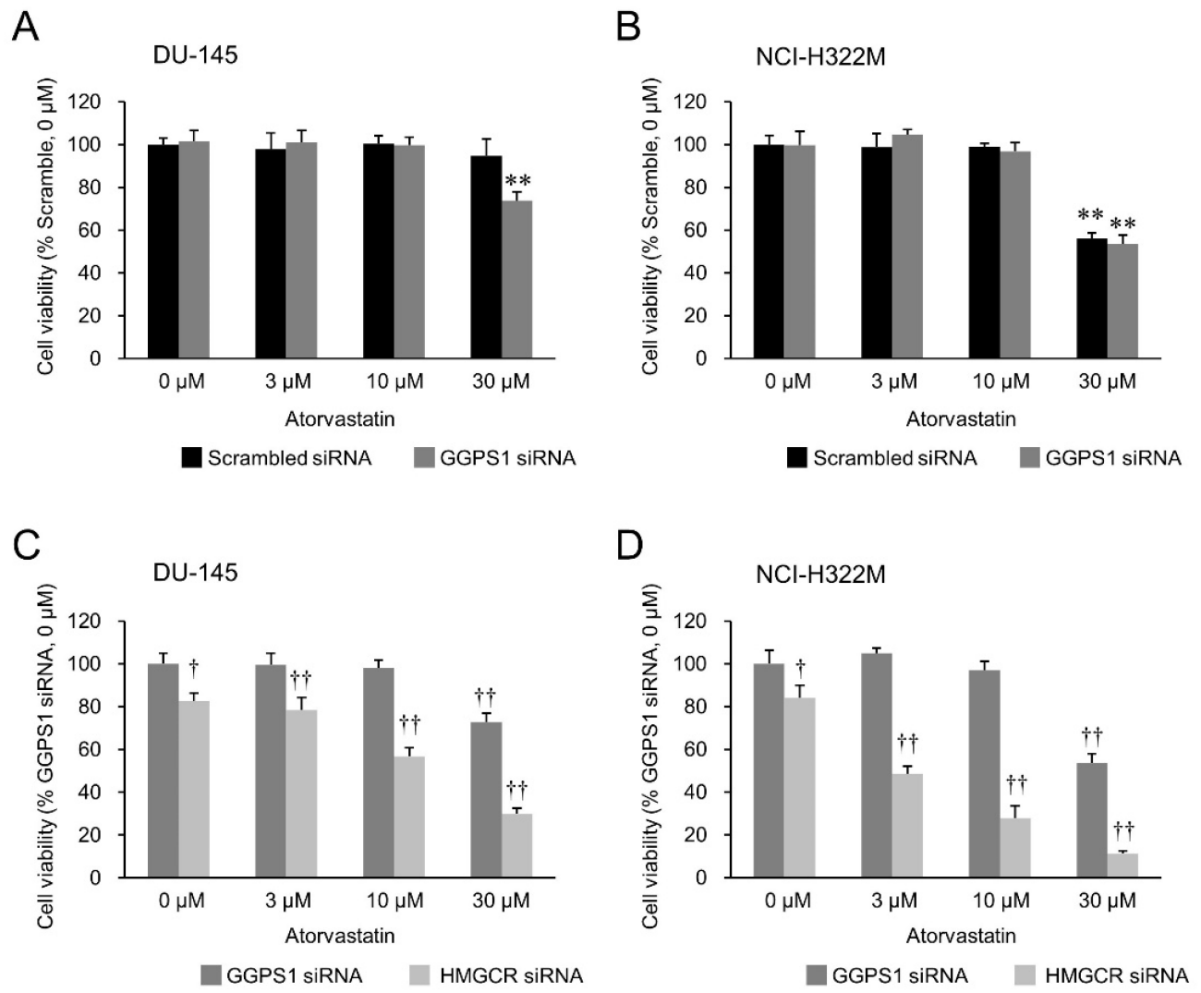
Effects of atorvastatin on HMGCR knockdown- versus GGPS1 knockdown statin-resistant cells Cell viability of the GGPS1 knockdown- or HMGCR knockdown-DU-145 cells **(A, C)** and NCI-H322M cells **(B, D)** treated with 0–30 μM atorvastatin 72 hours after the beginning of transfection. Values in scrambled siRNA-treated vehicle control cells (A, B) or GGPS1 siRNA-treated vehicle control cells (C, D) were set to 100%. Measurement values for each group were compared using the Bonferroni-Dunn *post-hoc* tests. Mean ± SD (*n* = 3) ^**^ p < 0.01 Comparison with scrambled siRNA-treated vehicle control cells, ^†^p < 0.05, ^††^p < 0.01 Comparison with GGPS1 siRNA-treated vehicle control cells.

Previous studies have shown that simultaneously targeting two enzymes of the mevalonate pathway via dual inhibition of HMGCR by fluvastatin and farnesyl pyrophosphate synthase using zoledronic acid (Figure 1) is more effective in inhibiting the growth of human pancreatic cancer cell lines than using fluvastatin alone [40]. Similarly, abrogation of 3-hydroxy-3-methylglutaryl coenzyme A synthase (HMG-CoA synthase) or GGPS1 (Figure 1) activity accentuated statin's growth inhibitory effect [38]. Therefore, we next tested if combination therapies affecting only the mevalonate pathway are able to overcome the relative statin resistance of epithelial or mixed epithelial-mesenchymal cancer cell lines. First, we examined the growth inhibitory effect of atorvastatin on the epithelial NCI-H322M (Supplementary Figure 1B) and mixed epithelial-mesenchymal DU-145 (Supplementary Figure 1D) cells when their GGPS1 expression was downregulated by siRNA knockdown. Downregulation of GGPS1 slightly improved the atorvastatin sensitivity of DU-145, but not NCI-H322M cells (Figure 5). In contrast, siRNA-mediated attenuation of HMGCR expression (Figure 2) provided a strong synergistic potentiation of atorvastatin's growth inhibitory effect in both of these relatively statin resistant cell lines (Figure 5).

## DISCUSSION

The metastatic cascade begins with an epithelial to mesenchymal transition (EMT), followed by invasion of detached cells or cell cluster through the basement membrane and intravasation into the vasculature. Cells that survive in the circulation reach distant sites and extravasate into the parenchyma, undergoing a mesenchymal to epithelial reverting transition (MErT) to integrate into the tissue as micrometastases [41]. Following a period of dormancy, which can last years or even decades, micrometastases undergo a second EMT and outgrow to form clinically evident metastases [42]. Distant micrometastases bear poor prognosis for cancer patients, with five-year survival rates ranging from 2-28% [43]. Preventing dissemination or micrometastatic outgrowth would delay this mortal stage in cancer progression. Unfortunately, at diagnosis of the primary tumor, many tumor cells may have already established dormant micrometastases [44]. The clinical challenge in targeting dormant micrometastases is that their quiescent cells exhibit chemoresistance to many standard therapies, which mostly target dividing cells [9]. Thus, there is a substantial clinical need for alternative therapies that either prevent metastasis initiation or suppress micrometastatic emergence.

Previously, analyses of drug sensitivity databases have indicated that mesenchymal phenotype [45] and enrichment for EMT features [28] are associated with higher sensitivity to statins in large panels of cancer cell lines across multiple tumor type, and experiments demonstrated that statins are candidate drugs for selectively targeting cells undergoing EMT [26–28]. We determined membrane E-cadherin to be a resistance marker for statin-mediated growth inhibition and demonstrated that the exogenous expression of membrane E-cadherin in a statin-sensitive cell line was sufficient to decrease statin potency [27]. We also developed a computational approach to predict drugs that may potentiate statin effects and identified several drugs that may orthogonally improve the growth inhibitory effect of statins in these cells [30]. However, we have not developed approaches to overcome the relative statin resistance of epithelial and mixed epithelial-mesenchymal cells that comprise dormant and reactivated micrometastases.

Here we show that downregulation of HMGCR expression by siRNA in epithelial NCI-H322M and mixed epithelial-mesenchymal DU-145 cells greatly improve their sensitivity to atorvastatin's growth inhibitory effect that is dependent on blocking downstream GGPP synthesis. The observed synergy implies that deeper suppression of GGPP synthesis may be required in these cell types than in mesenchymal cell-like tumor cells to prevent membrane translocation of Rho and Ras. Moreover, this may suggest that the reduction of both FPP and GGPP, as is obtained by blockade of HMGCR, is needed for suppression of cell growth. It has been well-established that FPP and GGPP modify different signaling G-proteins in tumor cells. For example, while Ras and Rheb are preferentially farnesylated, GGPP modification of Rac, RhoA, and Rab is required for their membrane localization and activity [46]. Moreover, the relative addiction to these G-proteins may vary based on tumor biology, suggesting differential sensitivity to sole depletion of either FPP or GGPP.

The effect of a drug on its target enzyme may be constrained by its innate inhibitory effect and its achievable extent of entry into its target cells in its active form. In theory, dual inhibition of the same target protein can ease the consequences of these limits, as has been shown for the attenuation of the oncogenic kinase, Bcr-Abl's activity [47]. Alternatively, downregulation of target protein expression is emerging as a viable therapy in neurodegenerative diseases, such as Huntington's Disease [48]. For HMGCR, this could also be achieved by targeting proteins that regulate the expression level of mevalonate pathway enzymes, such as the SREBP family- and Myc transcription factors, mTORC1, constituents of the PI3K-AKT pathway or AMPK (reviewed in Ref. [49]). Drugs available for this purpose include, metformin through its effect on AMPK, dipyrimadole through its effect on the transcription of mevalonate pathway enzymes, and other molecules (reviewed in Ref. [49]). Indeed, metformin can potentiate the growth inhibitory effect of statins on tumor cell lines and the growth of primary tumors in nude mice [50–52]. Future studies will test potential synergy between statins and metformin (and related compounds) at inhibiting primary tumor growth and metastatic outgrowth in murine tumor models. The synergistic effect of HMGCR's functional attenuation by simultaneously reducing its expression and its active site inhibition confirms the validity of a single enzyme-focused combination of these existing approaches.

## MATERIALS AND METHODS

### Cell culture

We selected four human cancer cell lines with different positions on the epithelial-to-mesenchymal spectrum, as characterized by their vimentin and E-cad expression profiles: epithelial NCI-H332M, mixed mesenchymal-epithelial DU-145, and mesenchymal PC-3 and HOP-92 cell lines (Supplementary Figure 1) [27]. The selected cell lines—lung cancer (HOP-92 and NCI-H322M) and prostate cancer-derived (PC-3 and DU-145)—were cultured in RPMI 1640 medium (Life Technologies, Grand Island, NY), supplemented with 10% heat-inactivated fetal bovine serum (HI-FBS, Life Technologies) and 1% penicillin/streptomycin (Life Technologies) at 37°C with 5% CO_2_.

### Gene silencing with siRNA

Predesigned siRNA oligonucleotides specific for HMGCR (NM_000859, siRNA ID#s142, targeted exon: 12, siRNA location: 1698), GGPS1 (NM_001037277, siRNA ID#s18107, targeted exon: 4, siRNA location: 653), and LDLR (NM_000527, siRNA ID#s4, targeted exon: 13, siRNA location: 2054) were obtained from Ambion (Austin, TX). The Silencer negative control siRNA (Ambion) was used as scrambled siRNA, a sequence provided by the manufacturer that has no significant homology to any gene. Reverse transfections were performed in 12-well dishes (6 × 10^4^ cells/mL) according to the manufacturer's instructions using Lipofectamine RNAiMax (Life Technologies), Opti-MEM (Life Technologies), and the siRNAs (final concentration 10 nM) for the respective targets. Cells (DU-145, NCI-H322M, PC-3, and HOP-92) were harvested 72 hours after the beginning of transfection for analysis of mRNA/protein expression and cell viability. Transfection efficiency was assessed by quantitative reverse transcription polymerase chain reaction (RT-PCR), western blotting, and immunofluorescence cytochemistry.

### Separation of total RNA and quantitative RT-PCR

Total cellular RNA was extracted from the siRNA-treated cells using an RNeasy mini kit (Qiagen, Hilden, Germany). Then, 1 μg of RNA was reverse transcribed into cDNA using the ReverTra Ace qPCR RT with gDNA Remover kit (Toyobo, Osaka, Japan). The cDNA was amplified through PCR with primer sets specific for the *HMGCR*, *GGPS1*, *LDLR*, and *Glyceraldehyde-3-phosphate dehydrogenase* (*GAPDH*) genes. The primer sets, the products of which include siRNA target sites, are shown in Supplementary Table 1. Real-time PCR was performed using a LightCycler rapid thermal cycler system (Roche Diagnostics, Lewes, UK) with LightCycler FastStart DNA MasterPLUS SYBR Green I mix (Roche Diagnostics). Each mRNA value was normalized to *GAPDH* mRNA.

### Western blotting

Cells were washed twice in cold phosphate buffered saline (PBS) and incubated with cold Pierce RIPA lysis buffer (Thermo Fisher Scientific, Rockford, IL) containing 1:100 Protease Inhibitor Cocktail (CalBiochem) for 5 minutes. Then, the cells were scraped, homogenized with a 27-gauge needle and vortexed at the highest setting for 1 min; the lysates were cleared by centrifuging at 16,000 *g* at 4 °C for 15 min. Protein concentration was determined with the bicinchoninic acid (BCA) method (BCA Protein Assay - Reducing Agent Compatible; Thermo Fisher Scientific). Protein extracts were boiled for 5 minutes and 10 μg of protein was loaded per lane. Proteins were separated on NuPAGE 4‒12% Bis Tris gel electrophoresis (Life Technologies), and transferred to a nitrocellulose membrane (iBlot Gel Transfer Stacks Nitrocellulose; Life Technologies) using iBlot Gel Transfer Device (Life Technologies). After blocking in 5% w/v non-fat dry milk for 1 hour, the membrane was probed with a mouse monoclonal anti-GGPS1 antibody (1:500, H00009453-M08, Abnova, Taiwan), or a rabbit polyclonal anti-HMGCR antibody (1:3000, PA5-37367, Thermo Fisher Scientific) for 1 hour at room temperature (RT). A rabbit monoclonal anti-GAPDH (1:1000; 14C10, Cell Signaling Technology, Danvers, MA) was used as a loading control. After washing with Tris-buffered saline with 0.1% Tween 20 (TBS-T), membranes were probed with a goat anti-mouse IgG-peroxidase (1:1000, R&D Systems, Minneapolis, MN) or a goat anti-rabbit IgG-peroxidase (1:5000, SeraCare Life Sciences, Milford, MA) as the secondary antibody for 1 hour at RT. This was followed by five washes in TBS-T and incubation in Clarity western ECL substrate chemiluminescent detection reagent (Bio-Rad, Hercules, CA) for 5 minutes prior to image acquisition. Protein bands were visualized using C-DiGit Blot Scanner (Li-Cor Biosciences, Lincoln, NE). Band quantification was done using ImageJ software to determine the integrated intensity of each band. Protein levels were normalized to GAPDH per lane. Statistical analysis was done using a two-way ANOVA, with significance level p < 0.05.

### Immunofluorescence microscopy

Cultured cells grown on coverslips in a 24-well plate were fixed with 2% paraformaldehyde (Nacalai Tesque, Kyoto, Japan) for 30 minutes, washed in PBS, and then permeabilized with 0.1% Triton-X-100 (Nacalai Tesque) prepared in PBS for 15 minutes. Following a PBS wash, non-specific proteins were blocked in 2% BSA for 15 minutes at RT. Cells were incubated with the following primary antibodies: a rabbit polyclonal antibody to HMGCR (1:80, ab98018, Abcam, Cambridge, UK), or a rabbit monoclonal antibody to LDLR (1:100, ab52818, Abcam) for 1 hour at RT. After washing with PBS, coverslips were incubated with CF-488A goat anti-rabbit IgG (1:200, Biotium, Hayward, CA) and 0.1 μM rhodamine-labeled phalloidin (Molecular Probes, Eugene, OR), a chemical which binds to F-actin, for 15 minutes at RT in the dark. Following a PBS wash, nuclei were stained with Hoechst 33342 (5 μg/ml) for 15 minutes at RT, washed, and mounted in an aqueous-based mounting medium, Fluoromount/Plus (Diagnostic Biosystems, Pleasanton, CA). Images were captured with a 60X oil immersion objective lens on a FluoView FV10i laser scanning confocal microscope (Olympus, Tokyo, Japan).

Visualization of endoplasmic reticulum (ER) was performed using CellLight ER-red fluorescent protein (ER-RFP), BacMam 2.0 (Thermo Fisher Scientific). Briefly, CellLight ER-RFP reagent was added to the cells (4 × 10^4^ cells/mL) in complete culture medium (1:80) and mixed gently. Then, cells were seeded on coverslips in a 24-well plate. Transduction of CellLight ER-RFP reagents occurred 4–6 hours after transfection. The cells were incubated for 24 hours, and then fixed with 2% paraformaldehyde (Nacalai Tesque) for 30 minutes. Immunocytochemistry was performed, as described above, using a rabbit polyclonal antibody to HMGCR (Abcam).

### Analysis of the effects of the siRNA treatment on cell number of the statin-resistant and statin-sensitive cells

Statin-resistant cells (DU-145 and NCI-H322M) and statin-sensitive (PC-3 and HOP-92) cells (6 × 10^4^ cells/mL) were cultured in medium containing 10 nM siRNA targeting *HMGCR*, *GGPS1*, or *LDLR* as described above. In the *HMGCR*- or *LDLR*-knockdown experiment, cell viability of each cell line was analyzed 72 hours after the beginning of transfection. In the *GGPS1*-knockdown experiment, cell viability was measured at 3, 6, and 9 days after the beginning of transfection. One-half of the medium containing 10 nM *GGPS1* siRNA was replaced every 3 days to maintain optimal culture conditions. After the incubation, the cells were harvested, and cell numbers were counted using a Scepter handheld automated cell counter (Millipore, Billerica, MA). The viability was determined by dividing the cell number of each experimental group by that of scrambled control cells. Viability of scrambled control cells was regarded as 100%.

### siRNA knockdown in cancer cell lines and testing their atorvastatin sensitivity

In 12-well dishes, cancer cells (6 × 10^4^ cells/mL) were cultured for 72 hours in the medium containing 10 nM siRNA targeting *HMGCR, GGPS1, or LDLR* as described above and various concentrations of atorvastatin (1–30 μM for statin-resistant cells and 0.1–3 μM for statin-sensitive cells, respectively). Cells treated with 0.3% DMSO served as vehicle control (0 μM). After the incubation, the cells were harvested, and cell numbers were counted using a Scepter handheld automated cell counter (Millipore). The viability was determined by dividing the cell number of each experimental group by that of scrambled siRNA-treated vehicle control cells (0 μM). Viability in each group of control cells was regarded as 100%. The data for each siRNA-treated group were compared with those for the controls (scrambled siRNA-treated group) using a student's two-tailed t-test or one-way ANOVA and Bonferroni-Dunn *post-hoc* tests. P values of less than 0.05 were considered statistically significant.

### Substrate rescue experiments in atorvastatin sensitive cells

To determine if metabolic intermediates of the mevalonate pathway or LDL treatment revert the cells’ atorvastatin-sensitive phenotype, the statin-sensitive cancer cell line HOP-92 was seeded in 12-well plates at a density of 1 × 10^5^ cells/mL (1 mL/well), incubated overnight, and then treated with 10 μM atorvastatin and various concentrations of R-mevalonic acid (10–50 μM; Sigma-Aldrich, St. Louis, MO), ubiquinone (25–200 μM; Wako, Osaka, Japan), dolichol (75–300 μM; Avanti Polar Lipids, Alabaster, AL), squalene (10–100 μM; Wako), FPP (1–25 μM; Echelon, Salt Lake City, UT), GGPP (1–25 μM; Sigma-Aldrich), or LDL (50–200 μg/mL; Alfa Aesar, Ward Hill, MA) for 48 hours. We chose the doses of each substrate according to published references [53, 54]. In select experiments, we photographed these cells with a phase-contrast microscope to capture any morphological changes. After the incubation, cells were harvested, and cell numbers were counted using a Scepter handheld automated cell counter (Millipore). Statistical analyses were performed using one-way ANOVA and Bonferroni-Dunn *post-hoc* tests. P values of less than 0.05 were considered statistically significant.

## SUPPLEMENTARY MATERIALS FIGURES AND TABLE



## Abbreviations

E-cad: E-cadherin
EMT: epithelial-to-mesenchymal transition
FPP: farnesyl pyrophosphate
GGPP: geranylgeranyl pyrophosphate
GGPS1: geranylgeranyl diphosphate synthase 1
HMGCR: HMG-CoA reductase
LDL: low-density lipoprotein
LDLR: LDL-receptors
MErT: mesenchymal-to-epithelial reverting transition.
