# Supplementary material for: Concomitant attenuation of HMG-CoA reductase expression potentiates the cancer cell growth-inhibitory effect of statins and expands their efficacy in tumor cells with epithelial characteristics
Source: Oncotarget. 2018 Jun 29;9(50):29304–15. doi: 10.18632/oncotarget.25448 (PMC6047681; doi:10.18632/oncotarget.25448)
Supplement: Supplementary file 1 [file oncotarget-09-29304-s001.pdf]

Concomitant attenuation of HMG-CoA reductase expression potentiates the cancer cell growth-inhibitory effect of statins and expands their efficacy in tumor cells with epithelial characteristics

SUPPLEMENTARY MATERIALS

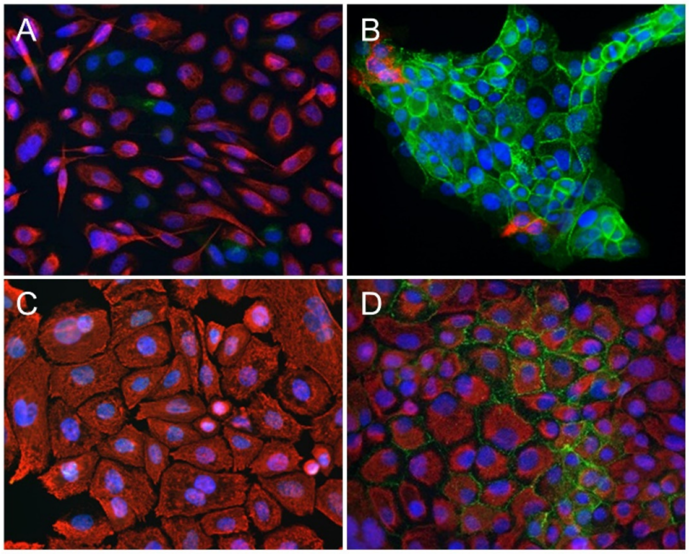

Supplementary Figure 1: E-cadherin and vimentin staining in prostate and lung cell lines. Representative merged images of **(A)** PC-3, **(B)** NCI-H322M, **(C)** HOP-92, and **(D)** DU-145 cell lines immunostained for vimentin (red, cytoplasmic), E-cadherin (green, membrane, cytoplasmic or nuclear), and Hoechst (blue, nucleus). Mesenchymal HOP-92 (Vim^{+}, nuclear E-cad only) and epithelial NCI-H332M (Vim^{-}, E-cad^{+}) (both lung cancer-derived) and mesenchymal PC-3 (Vim^{+}, E-cad^{-}) and mixed mesenchymal-epithelial DU-145 (Vim^{+}, E-cad^{+}) (both prostate cancer-derived) are shown.

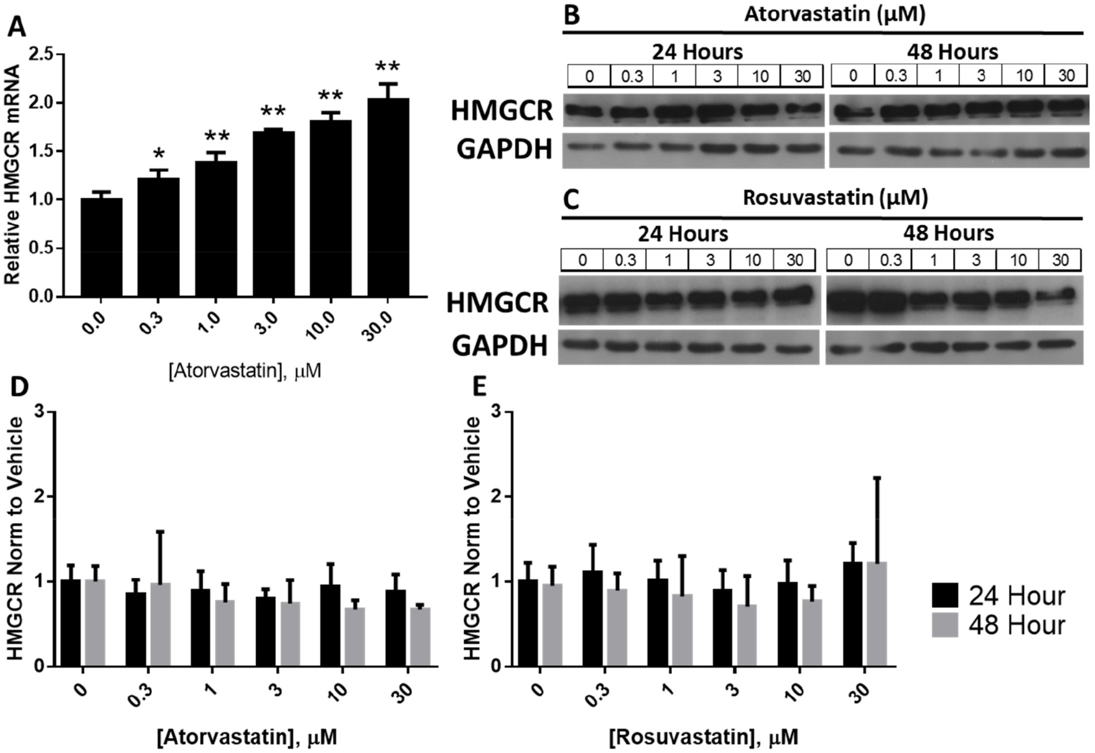

Supplementary Figure 2: HMGCR expression does not change upon atorvastatin or rosuvastatin treatment. HMGCR mRNA expression on DU-145 cells after 48 hours of atorvastatin treatment **(A)**. HMGCR expression in DU-145 after 24 hours or 48 hours of atorvastatin **(B)** or rosuvastatin **(C)** treatment at the indicated concentrations. HMGCR bands were quantified using densitometry and were normalized to GADPH at both time points for atorvastatin **(D)** and rosuvastatin **(E)** treatment. Results were normalized to vehicle. The results are representative of three independent experiments. Error bars represent the standard deviation (n = 3).

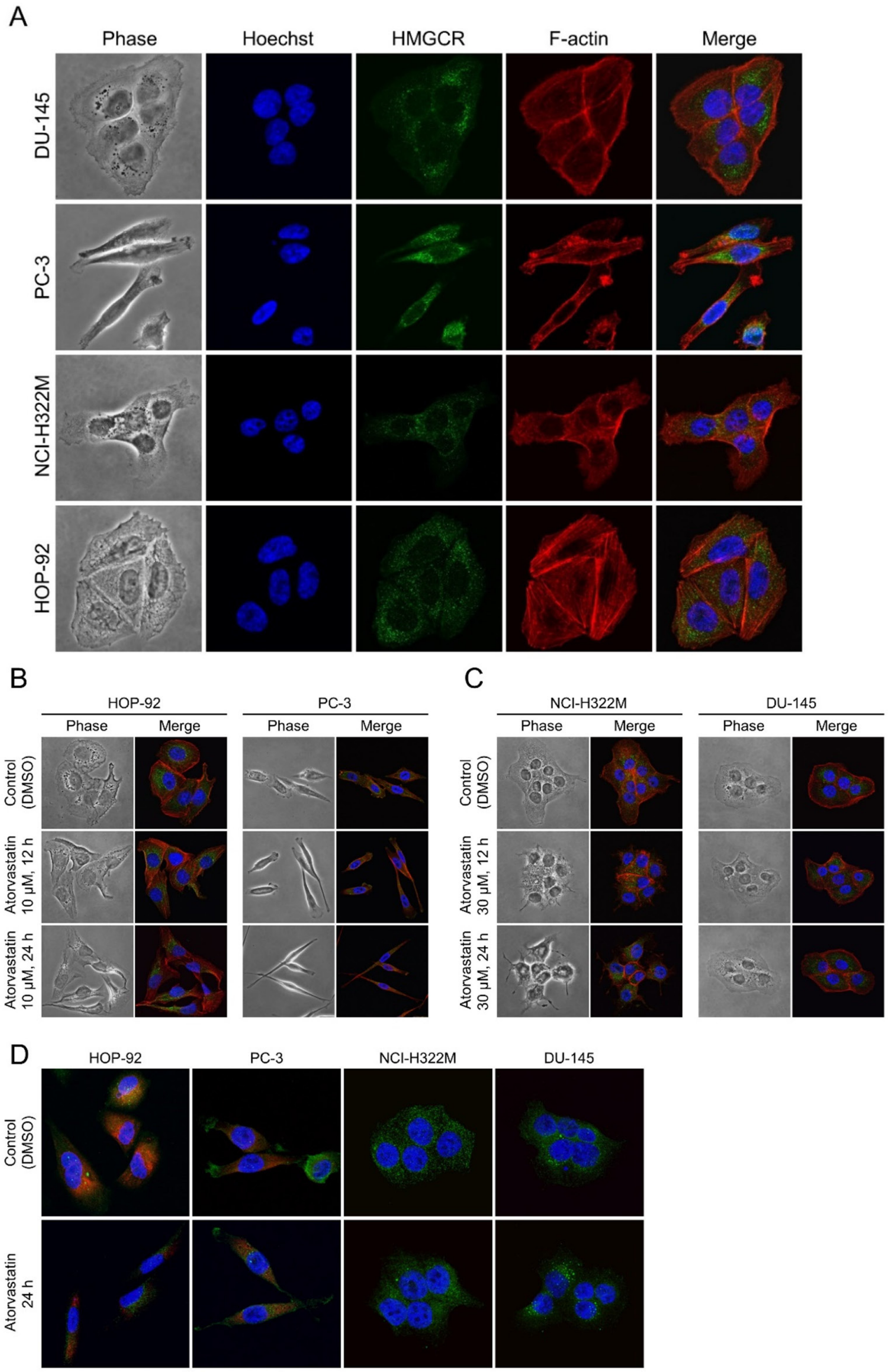

Supplementary Figure 3: Atorvastatin does not affect HMGCR's subcellular localization. (A) Images of the statin-sensitive cells (HOP-92, PC-3) and -resistant cells (NCI-H322M, DU-145) immunostained for HMGCR (green, perinuclear), F-actin (red, cytoplasmic), and Hoechst (blue, nucleus) prior to atorvastatin treatment. Localization of HMGCR in statin-sensitive cells **(B)** or -resistant cells **(C)** does not change after atorvastatin treatment for the indicated times and concentrations. **(D)** Localization of HMGCR and the endoplasmic reticulum (ER) marker protein, CellLight® ER-RFP (Red, perinuclear) also does not change in the cells treated with 10 μM (HOP-92, PC-3) or 30 μM (NCI-H322M, DU-145) atorvastatin for 24 hours. Note, however, that CellLight® ER-RFP expression was significantly less in DU-145 and NCI-H322M cell than in HOP-92 and PC-3 cells, perhaps due to less efficient transfection or ER-targeting efficiency.

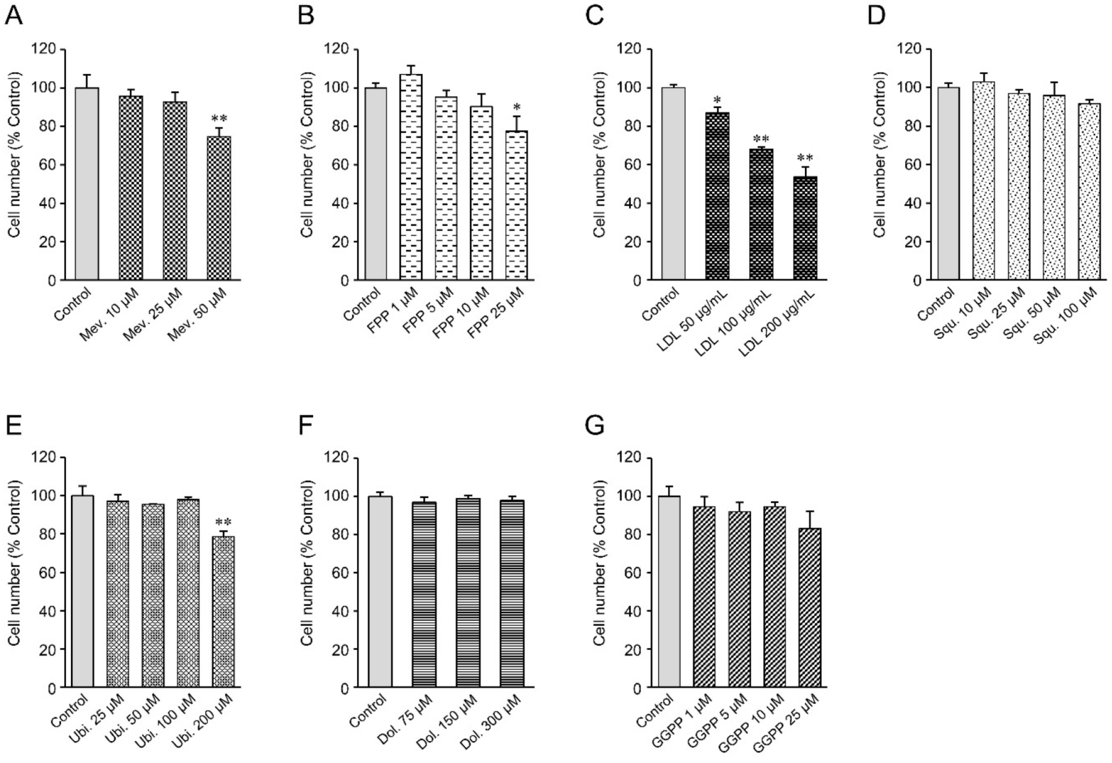

Supplementary Figure 4: Cell number of statin-sensitive HOP-92 cells treated with various intermediate metabolites of the mevalonate pathway. The HOP-92 cells were incubated with **(A)** mevalonate, **(B)** farnesyl pyrophosphate, **(C)** LDL, **(D)** squalene, **(E)** ubiquinone, **(F)** dolichol, and **(G)** geranylgeranyl pyrophosphate at the indicated concentrations. Cell number was measured at 48 hours after treatment. Cell number of vehicle-treated control was regarded as 100%. Measurement values for each group were compared using the Bonferroni-Dunn *post-hoc* tests. Mean ± SD (n = 3) * $p < 0.05$, ** $p < 0.01$ Comparison against control. Mev., mevalonate; FPP, farnesyl pyrophosphate; LDL, low-density lipoprotein; Squ., squalene; Ubi., ubiquinone; Dol., dolichol; GGPP, geranylgeranyl pyrophosphate.

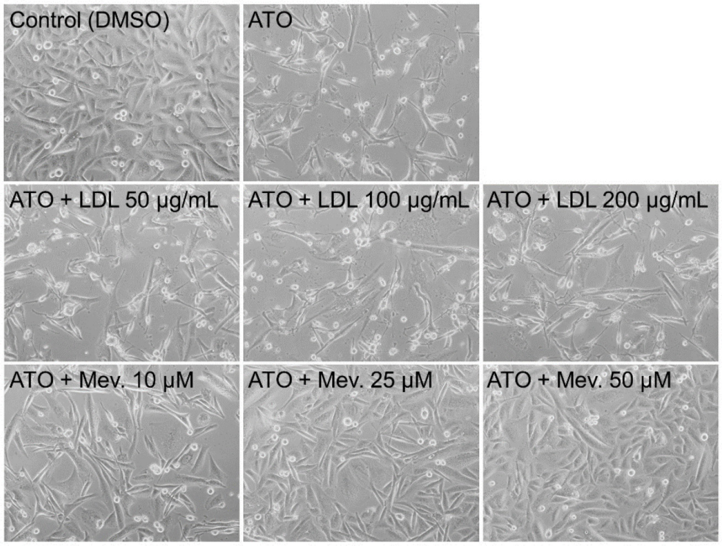

Supplementary Figure 5: LDL treatment does not rescue atorvastatin growth inhibition. Statin-sensitive HOP-92 cells were treated with 10 µM atorvastatin (ATO) for 48 hours and supplemented with LDL or mevalonate (Mev.) at the indicated concentrations. LDL supplementation did not rescue atorvastatin-mediated growth inhibition, while mevalonate supplementation provided protection.

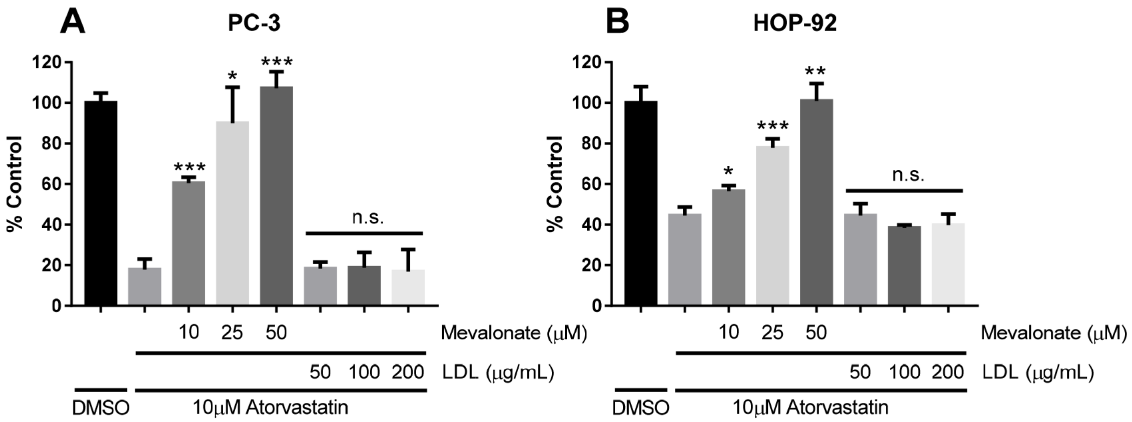

Supplementary Figure 6: LDL supplementation does not counter atorvastatin's growth inhibitory effect. LDL treatment does not rescue the growth inhibitory effect of atorvastatin in either, statin-sensitive PC-3 **(A)** or HOP-92 **(B)** cells. Cell number was determined 72 hours after statin treatment. Each column represents the mean ± SD (n = 3). The data are representative of three independent experiments. * $p < 0.05$, ** $p < 0.01$, *** $p < 0.001$. n.s. not significant. Mevalonate treatment was used as positive control for rescue effect.

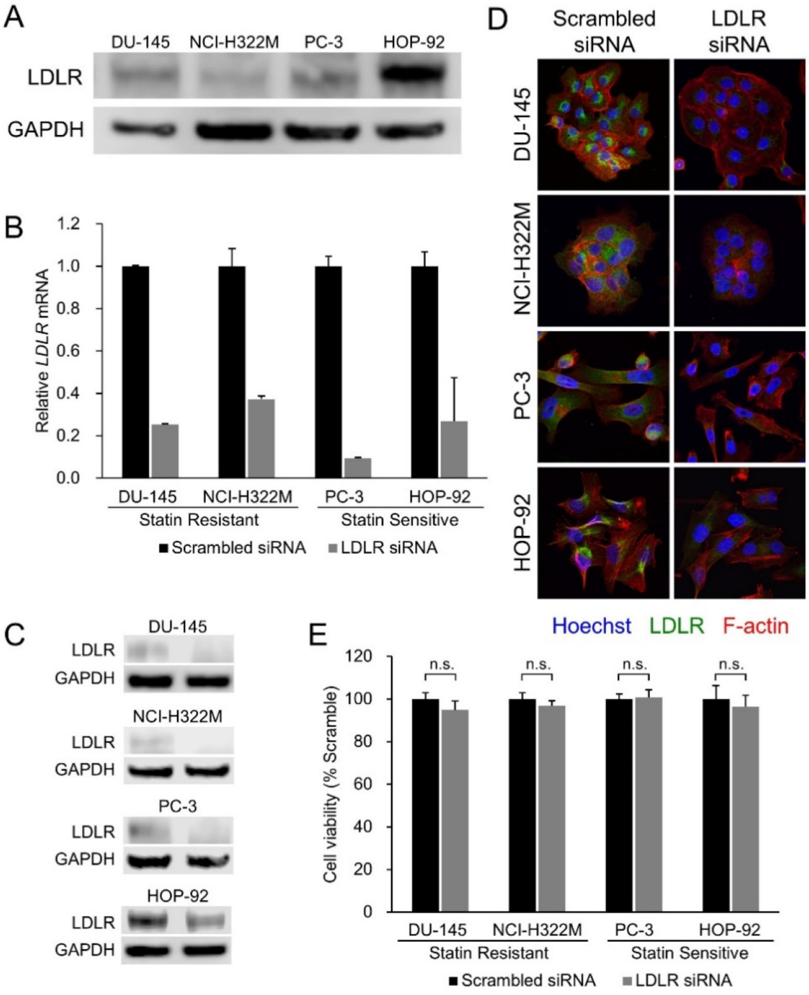

Supplementary Figure 7: LDLR knockdown does not affect cell viability. **(A)** The expression levels of LDL-receptor (LDLR) in the DU-145, NCI-H322M, and HOP-92 cell lines were determined by western blotting. GAPDH expression was used as the loading control. **(B)** Effect of siRNA transfection on *LDLR* mRNA levels in the four cell lines. The cells were transfected with 10 nM of siRNAs targeting *LDLR* or with their scrambled versions (control). Samples were analyzed 72 hours after the beginning of transfection. Data were normalized to the *GAPDH* mRNA levels in each sample and expressed in terms of a value relative to the control. Each column represents the mean ± SD (n = 3 for each group with triplicate determination). **(C)** Protein expression of LDLR was decreased after siRNA transfection as determined by western blotting. Left lane; scrambled control, Right lane; LDLR siRNA-treated cells. **(D)** Merged images of the siRNA-transfected cell lines immunostained for LDLR (green, perinuclear), F-actin (red, cytoplasmic), and Hoechst (blue, nucleus). LDLR immunoexpression was diminished by LDLR siRNA treatment. **(E)** Cell viability of the statin-resistant and -sensitive cell lines treated with LDLR siRNA 72 hours after the beginning of transfection. Values in scrambled control were set to 100%. Each value represents the mean ± SD (n = 3). Data were analyzed using a student's two tailed t-test with a significance level of $p < 0.05$. n.s. not significant.

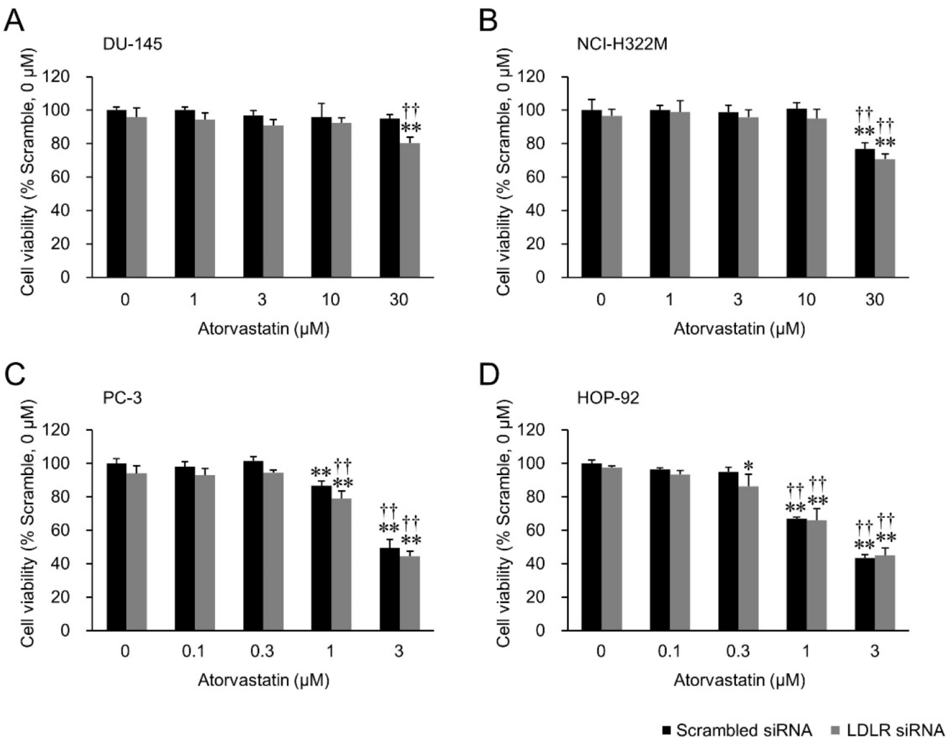

Supplementary Figure 8: Effects of atorvastatin on LDLR knockdown statin-resistant and -sensitive cells. Cell viability of the LDLR knockdown-DU-145 cells **(A)**, -NCI-H322M cells **(B)**, -PC-3 cells **(C)**, and -HOP-92 cells **(D)** treated with atorvastatin 72 hours after the beginning of transfection. Values in scrambled siRNA-treated vehicle control cells (0 μM) were set to 100%. Measurement values for each group were compared using the Bonferroni-Dunn *post-hoc* tests. Each value represents the mean ± SD (n = 3). * $p < 0.05$, ** $p < 0.01$ Comparison with scrambled siRNA-treated vehicle control cells. ††$p < 0.01$ Comparison with LDLR siRNA-treated vehicle control cells.

Supplementary Table 1: Forward and reverse primer sequences for RT-PCR

Gene	Primer sequence	Product size (bp)
HMGCR	5′- CCCAGCCTACAAGTTGGAAA -3′	152
	5′- AACAAGCTCCCATCACCAAG -3′	
GGPS1	5′- CACTTGGGCTCTTTTTCCAA -3′	170
	5′- GCGCAAGATATTCTGCACCT -3′	
LDLR	5′- TCACTCCATCTCAAGCATCG -3′	268
	5′- GGTGGTCCTCTCACACCAGT -3′	
GAPDH	5′- GAGTCAACGGATTTGGTCGT -3′	238
	5′- TTGATTTTGGAGGGATCTCG -3′	
